# Special issue on digital era education: tracing digital health transformation in women’s health nursing

**DOI:** 10.4069/kjwhn.2023.09.15

**Published:** 2023-09-26

**Authors:** Sook Jung Kang

**Affiliations:** College of Nursing, Ewha Womans University, Seoul, Korea

The coronavirus disease 2019 (COVID-19) outbreak started in December, 2019 in the Chinese city, Wuhan. As of September 2023, we are emerging from the COVID-19 pandemic, and it has changed our health care settings as well as nursing care environments. One of the biggest changes from the pandemic is that the digital era has come to our daily life and has affected nursing care, nursing education, and research. However, did the digital era come due to the pandemic? Many researchers and nurses before and right after the COVID-19 pandemic had foreseen that digital technology or telemedicine will be one of the main issues for nursing practice, education, and research in our current era. Jeong [1] emphasized the usefulness of artificial intelligence, machine learning, and deep learning and pushed for educating nurses in clinical settings and incorporating digital technology in the nursing curriculum. While COVID-19 may have accelerated this transformation process, the digital technology was already coming to our field of health care.

*The Korean Journal of Women Health Nursing* is publishing this special issue of ‘digital era education for women’s health and well-being’ because now is the time to reflect on the changes introduced by widespread digital technology and assess unmet needs relating to women’s health. We are also challenged to rethink our expectations of what we can do with digital technology and what need to be done with further. Recognizing the importance of digital technologies in shaping the future of global health, the World Health Organization’s “Global Strategy on Digital Health 2020–2025” outlines principles and key components of digital health care and stated that countries around the world need to be prepared and equipped with digital health skills [2]. Recently, the International Council of Nurses has issued position statement about digital health transformation and nursing practice [3]. The position statement, announced on September 1, 2023, defines digital health as “the field of knowledge and practice associated with the development and use of digital technologies,” which is beyond the concept of e-health. The statement enumerates important recommendations in terms of global health nursing and responsibilities of national nurses’ associations, as well as what to expect for nurses, nurse educators, researchers, and policy influencers [2].

This special issue contains evidence of the digital transformation for women’s health, including women with cancer [4] and older adults [5], critical appraisal of mobile apps for pregnant women [6] and women’s health education using YouTube [7], and presents new challenges to incorporate artificial intelligence into simulation [8]. However, more evidence regarding interventions and frameworks and/or models that can enhance rigor of digital health-incorporated research is needed. Also, since digital health can raise the issue of ethical concerns regarding health equity, increased workload for nurses, and information safety, the specific ramifications of those issues in relation to women’s health require further dialogue and study. Finally, as digital health is rapidly evolving, support of up-to-date education and training is needed for nurses and nursing students so that they are empowered and competent for the next step of trans-digital health care settings.

We hope you enjoy this special issue and share your comments and critiques through letters to the Editor.
